# hsa-miR-199b-3p Prevents the Epithelial-Mesenchymal Transition and Dysfunction of the Renal Tubule by Regulating E-cadherin through Targeting KDM6A in Diabetic Nephropathy

**DOI:** 10.1155/2021/8814163

**Published:** 2021-06-27

**Authors:** Shoujun Bai, Xiaoyan Xiong, Bo Tang, Tingting Ji, Xiaoying Li, Xiaolei Qu, Weiliang Li

**Affiliations:** ^1^Department of Nephrology, Qingpu Branch of Zhongshan Hospital Affiliated to Fudan University, 1158 Gongyuan East Road, Qingpu District, Shanghai 201700, China; ^2^Department of Urology, Qingpu Branch of Zhongshan Hospital Affiliated to Fudan University, 1158 Gongyuan East Road, Qingpu District, Shanghai 201700, China

## Abstract

Diabetic nephropathy (DN) is the leading cause of end-stage renal disease. The association between epithelial-mesenchymal transition (EMT) and fibrosis is quite ascertained, but its link to eventual tubule dysfunction is missing. Here, we show that human microRNA- (hsa-miR-) 199b-3p protects renal tubules from diabetic-induced injury by repressing KDM6A, a histone lysine demethylase regulating E-cadherin expression. Lower E-cadherin expression is related to a higher level of KDM6A, while E-cadherin is promoted upon treatment with the KDM6A inhibitor GSK-J4 in both high glucose- (HG-) induced HK2 cells and the kidneys from streptozotocin- (STZ-) induced type 1 diabetic mice. However, overexpression or RNA silencing of E-cadherin fails to alter KDM6A expression. We also show that the upregulation of KDM6A is associated with the increased methylation level of the E-cadherin promoter. Then, the target prediction results and a dual-luciferase assay show that hsa-miR-199b-3p is a new miRNA that targets KDM6A. Overexpression of hsa-miR-199b-3p increases E-cadherin expression and prevents EMT through repressing KDM6A expression in HG-induced HK2 cells. In contrast, inhibitor-induced hsa-miR-199b-3p knockdown has opposite effects, as it decreases E-cadherin level and worsens EMT, accompanied by increased levels of KDM6A. Besides, Mir199b-knockout mice without mmu-miR-119b-3p expression exhibit more renal tubule dysfunction and more serious kidney tissue damage upon treatment with STZ. These results demonstrate that hsa-miR-199b-3p improves E-cadherin expression and prevents the progression of DN through targeting KDM6A. miR-199b-3p could be a future biomarker or target for the diagnosis or treatment of DN.

## 1. Introduction

Diabetic nephropathy (DN) is a serious microvascular complication of diabetes and a common cause of end-stage renal disease [1, 2]. With the increasing number of new diagnoses and a 5-year survival rate of ~20%, DN has attracted much attention [3]. The common damage of DN is glomerular injury and tubular injury [4–6]. Some researchers think that the proximal tubule injury critically promotes renal injury and even the initial stage of DN [7, 8]. In recent years, many researchers have indicated that epithelial-mesenchymal transition (EMT), a main pathological process of renal tubular epithelial cells, promotes renal tubule dysfunction, tubulointerstitial fibrosis, and tubular atrophy which are the classical feature of end-stage renal disease. [9–12].

While most studies have focused on the role of the TGF-*β*/BMP7/Smad pathways in the DN progression, recent studies have indicated that chromatin modifications may also significantly affect the DN occurrence [13–16]. KDM6B (also known as JMJD3, jumonji domain-containing 3) and KDM6A (also known as the histone demethylase UTX (ubiquitously transcribed tetratricopeptide repeat, X chromosome)), which was identified in 2007 [17], can specifically remove di- and trimethyl groups from the lysine 27 residue of histone H3 (H3K27) [18, 19]. In this way, ectopic expression of KDM6A is closely associated with altered gene expression. Interestingly, several reports have shown that KDM6A plays a novel regulatory role in diabetic kidney disease. Chen et al. found that KDM6A can regulate inflammation and DNA damage in db/db mice [20]. Lin et al. found that KDM6A can aggravate diabetic kidney injury by inducing podocyte dysfunction [21]. However, little investigation to expound the role of KDM6A in renal tubular injury in DN has been carried out.

MicroRNAs (miRNAs) are endogenous noncoding RNAs 21-23 nucleotides (nt) in length that can regulate gene expression by binding to the 3′ untranslated regions (3′UTR) of target gene mRNA [22]. Recent advances in the study of miRNAs have shown that miRNAs can control EMT and renal tubule dysfunction in renal disease, indicating that miRNAs may have a potent effect on DN. For example, hsa-miR-21 and hsa-miR-302 show prominent activity in regulating the process of EMT and fibrosis in DN [23, 24]. We speculate that a miRNA participates in the progression of DN by targeting KDM6A mRNA.

In this study, we report that hsa-miR-199b-3p can alleviate the process of renal tubule EMT and tubule dysfunction in DN through binding to KMD6A mRNA. We also explore the deleterious effect of KDM6A in aggravating renal tubule injury by repressing E-cadherin expression.

## 2. Materials and Methods

### 2.1. Cell Cultures, Reagents, and Transfections

HK2 cells were obtained from the China Center for Type Culture Collection (CCTCC) and cultured in MEM (5 mM D-glucose) (Gibco) supplemented with 10% FBS (Gibco), 100 *μ*g/mL streptomycin, and 100 U/mL penicillin (HyClone) at 37°C with 5% CO_2_. For EMT induction, HK2 cells were grown in high-glucose (HG) medium (30 mM D-glucose) or high-mannitol (Mann) medium (5 mM D-glucose and 25 mM mannitol) for 72 h. Streptozotocin (STZ) and GSK-J4 were from Sigma.

For HK2-E-cadherin stable cell line construction, HK2 cells were transduced with lentivirus encoding or not E-cadherin sequence and selected with puromycin (Sigma, 2 *μ*g/mL) for two weeks. Lentivirus packaging was performed in HEK293T cotransfected with psPAX2, pMD2G, and pCDH-CMV-Mcs-Ef1-Puro (Addgene) for 72 h. HK2 cells were transduced with lentivirus encoding miR-199b-3p or miNC to generate HK2-miRNA stable cells, as described [25].

The small interfering RNAs (siRNAs) for negative control (si-NC) or for downregulating E-cadherin (si-E-cadherin) were obtained from Invitrogen. The miR-199b-3p inhibitor (inhibitor) and negative inhibitor control (inhibitor NC) were obtained from RiboBio. Transfection experiments were performed with the Lipofectamine 2000 reagent according to the manufacturer's instruction (Thermo Fisher Scientific).

The sequences of siRNA and miRNA are exhibited in Table S2.

### 2.2. Wound Healing Assay

The cells were seeded at the density of 5 × 10^5^ cells/well in 6-well plates. The cell monolayer was disrupted with a 200 *μ*L pipette tip to form a “wound.” Photographs were taken at 0 h and 48 h. The wound areas were measured by using ImageJ software, and the “relative wound area” was normalized against wound areas at 0 h.

### 2.3. Animals, Diabetic Animal Models, and GSK-J4 Treatment

12-week-old male C57BL/6 mice (Guangdong Medical Laboratory Animal Center) were intraperitoneally injected with 190 mg/kg STZ dissolved in 0.01 mol/L citrate buffer (pH 4.5) or equivalently citrate buffer to induce diabetes. The blood glucose levels were equalized by adding 1-2 unit/kg insulin in each diabetic mouse as described previously [21]. Mice with fasting blood glucose > 200 mg/dL were used as diabetics. For GSK-J4 treatment, mice were subcutaneously injected with 0.4 mg/kg each day for 1 week after the onset of diabetes. miR-199b-knockout C57BL/6 mice were obtained through the CRISPR/Cas9 system performed by the Nanjing Biomedical Research Institute (NBIR) of Nanjing University, China.

### 2.4. Mouse Metabolic Measurements

The albumin and creatinine (Cr) ratio (ACR) was determined with the mouse albumin ELISA kit (Bethyl Laboratories) and Creatinine Assay Kit (BioAssay System). Blood urea nitrogen (BUN) was tested by using the Urea Assay Kit (BioAssay System). Proteinuria was examined by using the Proteinuria ELISA Kit (Nanjing Anyan Biological Technology). All of those assays were performed at 8 weeks after the onset of diabetes in each mouse.

### 2.5. Quantitative Real-Time Polymerase Chain Reaction (qRT-PCR)

Total RNA was extracted using the TRIzol reagent (Invitrogen), and cDNAs were synthesized using the PrimeScript RT reagent kit (TaKaRa) except that of miRNA by TaqMan Advanced miRNA cDNA synthesis kit (Waltham). PCR quantification was performed as previously described [26]. To test miRNA expression, we used stem-loop qRT-PCR for miR-199a-3p, miR-199b-3p, and miR-758-3p following the protocol described previously [27, 28]. Primers are shown in Table S2.

### 2.6. Protein Extraction and Western Blot Analysis

Proteins were extracted from cells (RIPA, P0013E, Beyotime Biotechnology) and mouse kidney (One Step Animal Tissue Active Protein Extraction Kit, C500006-0020, Sangon Biotech), and the protein lysates quantified through a bicinchoninic acid assay kit (Boster). Then, the proteins were boiled for 5 min at 100°C in loading buffer (20 *μ*g) and fractionated in SDS-PAGE gel at 80 V for 30 min and then 100 V for 1 h. Next, the proteins were transferred to a nitrocellulose membrane (Boster) at 300A for 1.5 h. The membranes were blocked in Tris-buffered saline containing 0.1% Tween 20 (TBST) and 5% fat-free milk for 1 h. Then, the membranes were incubated with primary antibodies overnight at 4°C. After washing, the membranes were incubated with secondary antibodies for 2 h at room temperature. The membranes were incubated with a high-sig electrochemiluminescence kit (FDbio Science) and detected by using an image analysis system (Image-Pro Plus 6.0, Media Cybernetics).

All primary antibodies except anti-H3K27me2 (Invitrogen) and anti-H3K27me3 (Invitrogen) were from Abcam, and all secondary antibodies were from Sino Biological.

### 2.7. Dual-Luciferase Assay

HEK293T cells were transfected with pMIR-KDM6A 3′-UTR and pMIR-KDM6A 3′-UTR mutant plasmid (500 ng) accompanied by a pRL-TK plasmid (50 ng) (Promega), respectively. WT KDM6A 3′-UTR: gcAACAUCUUACAGACUACUGa; mutant KDM6A 3′-UTR-Mut: gcAACUACUCAAGACACAAGCa. At the same time, miR-199b-3p mimics or negative control (150 nM) was cotransfected with those plasmids. Luciferase activity was examined using the Dual-Luciferase Reporter Assay System (Promega) at 48 h after transfection [29].

### 2.8. Tissue Histochemical Staining, HE, and PAS Staining

Mouse kidneys were obtained from 20-week-old mice at 8 weeks after the onset of diabetes. Mouse kidneys embedded in paraffin were cut into 4 *μ*m thick sections and stained with Periodic Acid-Schiff (PAS) and Hematoxylin Eosin (HE) (Sigma). An experienced pathologist analyzed stained slides for histopathological examination under light microscopy. For histochemical staining, paraffin-embedded sections were stained with anti-KDM6A, anti-E-cadherin, and anti-TGF*β* as described previously [30]. The relative expression was quantified by using ImageJ. AOD = IOD/area. “IOD” means the total brown signal strength of the histochemical staining region, and the “area” represents the total pixels or area of the histochemical staining region. So the “AOD” means the average brown signal strength in a single-pixel or unit area.

### 2.9. Methylation-Specific PCR (MS-PCR)

Genomic DNA was isolated from cultured HK2 cells using the PureLink™ Genomic DNA Mini Kit (Invitrogen). For each sample, 1 *μ*g of DNA was used for the bisulfite transformation by using EpiTect Plus DNA Bisulfite Kit (Qiagen) according to the manufacturer's protocol. The unmethylated-specific (U) primers and methylated-specific (M) primers are shown in Table S2. According to others' work, the primers were designed using the MethPrimer database [31, 32]. The PCR reaction mixture (20 *μ*L) contained 80 ng of bisulfite-treated DNA and 0.5 *μ*M of each primer in 1X EpiTect Master Mix. PCR conditions were as follows: step 1: 95°C, 10 min; step2: 35 cycles of 94°C for 15 s, 50°C for 30 s, and 72°C for 30 s; and step3: finally 72°C for 10 min.

### 2.10. Chromatin Immunoprecipitation- (ChIP-) qPCR Analysis

ChIP was performed using ChIP Kit-One Step (Abcam) according to the manufacturer's protocol. 2 *μ*L of anti-H3K27me2 (Invitrogen) or anti-H3K27me3 (Invitrogen) was used in the ChIP process. 2 *μ*L of eluted DNA from ChIP was used in a 20 *μ*L PCR reaction. The qPCR assay was performed as described above. The primers are shown in Table S2.

### 2.11. Statistical Analysis

The study's data were analyzed by the Student's *t*-test using Prism (version 5; GraphPad Software). Comparisons of multiple groups were performed using 1-way ANOVA followed by Tukey's multiple comparison test. All data were assessed for normality of distribution using the Shapiro-Wilk test. Results are expressed as the mean ± SD. Values were considered statistically significant if *P* < 0.05.

## 3. Results

### 3.1. KDM6A Promotes EMT in HK2 Cells

**Figure 1:**
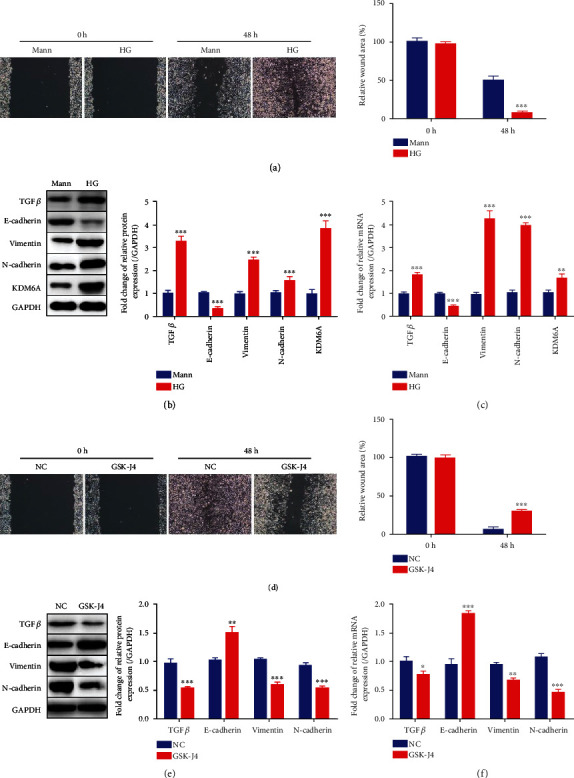
The promotive role of KDM6A in regulating EMT in HK2 cells. (a) Representative images of migration and the graphs of wound area quantification in HK2 cells cultured in HG or Mann at 0 h and 48 h. *n* = 10. (b) Western blot analysis of TGF*β*, E-cadherin, vimentin, N-cadherin, and KDM6A in HK2 cells cultured in HG or Mann for 72 h. *n* = 3. (c) The relative level of TGF*β*, E-cadherin, vimentin, N-cadherin, and KDM6A expression by qPCR in HK2 cells cultured in HG or Mann for 72 h. *n* = 3. (d) Representative images of migration and the graphs of wound area quantification in HK2 cells cultured in HG treatment with GSK-J4 (40*μ*M) or equivoluminal ddH_2_O (NC) at 0 h and 48 h. *n* = 10. (e) Western blot analysis of TGF*β*, vimentin, N-cadherin, and E-cadherin in HK2 cells cultured in HG treatment with GSK-J4 or ddH_2_O for 72 h. *n* = 3. (f) The relative level of TGF*β*, vimentin, N-cadherin, and E-cadherin expression by qPCR in HK2 cells cultured in HG treatment with GSK-J4 or ddH_2_O for 72 h. *n* = 3. Mean ± standard error of the mean values is presented. ^∗^*P* < 0.05, ^∗∗^*P* < 0.01, and ^∗∗∗^*P* < 0.005 (Student's *t*-test).

We first investigated the expression and significance of KDM6A in HK2 cells cultured in HG- or Mann-containing medium. We confirmed the change in HK2 cell migration, closely associated with EMT, in the HG group [33]. Relative to the Mann-induced HK2 cells, HG-induced HK2 cells showed significantly increased migration, suggesting that HK2 cells underwent substantial EMT (Figure 1(a)). The EMT markers, such as TGF*β*, E-cadherin, vimentin, and N-cadherin, were detected by immunoblotting and qPCR. The immunoblotting results showed decreased E-cadherin and increased TGF*β*, vimentin, and N-cadherin expression in HG-induced HK2 cells compared to Mann-induced HK2 cells (Figure 1(b)), consistent with the qPCR results (Figure 1(c)). Interestingly, the expression of KDM6A in the HG group was also elevated at both the transcriptional and translational levels (Figures 1(b) and 1(c)), indicating that upregulation of KDM6A may be associated with the EMT progression of HK2 cells induced by HG.

To confirm this hypothesis, GSK-J4, a KDM6A inhibitor, was utilized to treat HK2 cells in HG medium (GSK-J4 group), and another group of HK2 cells was treated with negative control (NC group). As detected by the wound healing assay, migration was decreased in the HK2 cells treated with GSK-J4 (Figure 1(d)). Increased E-cadherin expression and decreased expression of TGF*β*, vimentin, and N-cadherin in the GSK-J4 group were observed by western blotting and qPCR, indicating that the inhibition of KDM6A could reduce the degree of EMT in HG-induced HK2 cells (Figures 1(e) and 1(f)).

These results illustrate that KDM6A may promote the progression of EMT in renal tubular epithelial cells.

### 3.2. KDM6A Plays a Pivotal Role in Kidney Injury in DN

**Figure 2:**
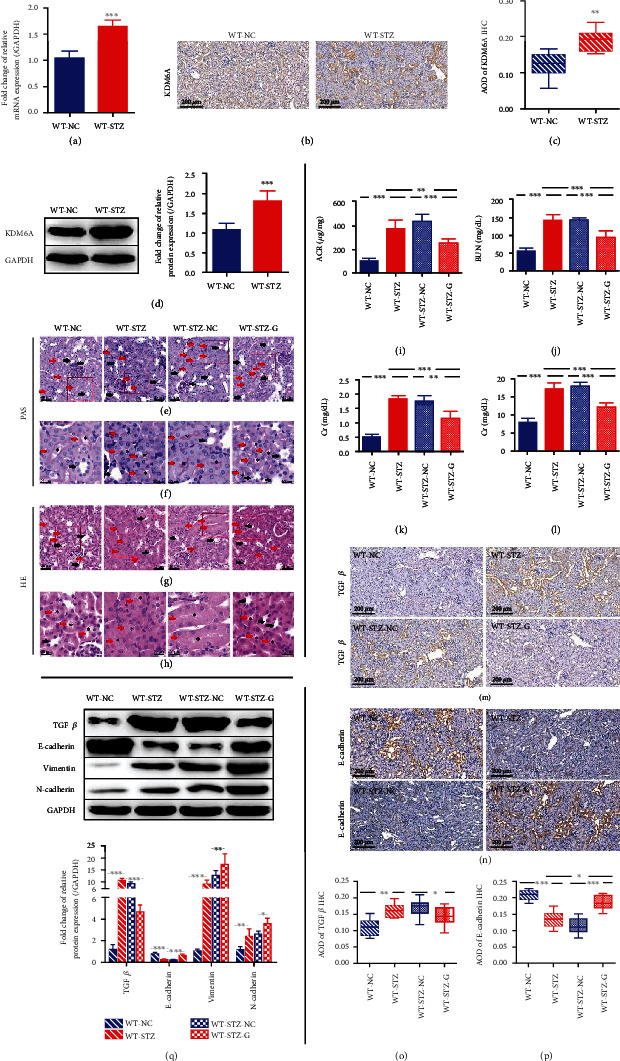
The pivotal role of KDM6A in the progression of kidney injury in DN. (a) The relative level of KDM6A expression by qPCR in kidney tissue from C57BL/6 mice (WT-NC) and STZ-induced mice (WT-STZ) (at 8 weeks after the onset of diabetes). *n* = 3. (b) Representative images of KDM6A immunohistochemical staining in kidneys from WT-NC and WT-STZ. Scale bar, 200 *μ*m. (c) The graphs showing quantification (AOD = IOD/area) of KDM6A immunohistochemical staining results in (b). *n* = 10. (d) Western blot analysis of KDM6A in the kidney from mice. *n* = 6. (e, g) Representative images of PAS staining and HE staining of kidneys from C57BL/6 mice with different treatments. STZ-induced C57BL/6 mice treated without (WT-STZ-NC) or with GSK-J4 (WT-STZ-G). Scale bar, 40 *μ*m. Proximal tubule marked with red arrows and distal tubule marked with black arrows. (f, h) Representative images of area amplification marked with red boxes in (e) and (g), respectively. Scale bar, 20 *μ*m. Proximal tubule marked with red arrows and distal tubule marked with black arrows. Lumen of proximal tubules marked with black asterisk. (i–l) The graphs showing the results of ACR, BUN, Cr, and proteinuria in different treated mice at 8 weeks after the onset of diabetes. *n* = 6. (m, n) Representative images of TGF*β* and E-cadherin immunohistochemical staining in kidneys of different treated mice. Scale bar, 50 *μ*m. (o, p) The graphs showing quantification (AOD = IOD/area) of TGF*β* and E-cadherin immunohistochemical staining results in (l) and (m), respectively. *n* = 10. (q) Western blot analysis of TGF*β*, E-cadherin, vimentin, and N-cadherin in the kidney from mice. *n* = 6. Mean ± standard error of the mean values is presented. ^∗^*P* < 0.05, ^∗∗^*P* < 0.01, and ^∗∗∗^*P* < 0.005 (Student's *t*-test in (a), (c), and (d); one-way ANOVA in (i)–(l) and (o)–(q).

Based on the *in vitro* results, we next investigated the role of KDM6A in DN *in vivo* using an STZ-induced mouse model and control mice (the WT-STZ and WT-NC groups, respectively). We indeed found a higher KDM6A expression in the kidney tissues of diabetic mice induced with STZ for 12 weeks relative to the control group by qPCR, immunohistochemistry (IHC), and western blot (Figures 2(a)–2(d)). To further explore the influence of KDM6A on the progression of diabetic kidney injury, GSK-J4 was applied to treat the diabetic mice, and some diabetic mice acted as a negative control group (the WT-STZ-G and WT-STZ-NC groups, respectively). Diabetic kidney injury was evaluated by assessing the renal proximal tubules' morphology and distal renal tubules associated with tubular injury. HE and PAS staining of the kidneys of STZ-induced mice showed obvious progressive renal proximal tubular dilatation, a thickened and irregular tubular basement membrane, and a reduced proximal tubule lumen characteristic of tubular injury relative to that of control mice, which were associated with tubulointerstitial fibrosis [34, 35] (Figures 2(e)–2(h)). However, the degree of proximal tubule injury in the diabetic mice was dramatically decreased after treatment with GKS-J4 (Figures 2(e)–2(h)). Several typical pathological characteristics of DN (the ACR, BUN, Cr, and proteinuria) were then measured, indicating tubular dysfunction or kidney injury [10, 36–38]. These characteristics were aggravated in STZ-induced mice but partly restored by GSK-J4 treatment (Figures 2(i)–2(l)), consistent with the HE and PAS staining results. These results indicated that the downregulation of KMD6A might alleviate kidney injury in DN mice.

Interestingly, while the quantitative IHC and western blot results revealed suppressed expression of E-cadherin and elevated expression of TGF-*β* in STZ-induced diabetic mice, the expression of both was altered by GSK-J4 (Figures 2(m)–2(q)). After treating the diabetic mice with GSK-J4, the decreased E-cadherin level was restored, and the increased TGF-*β* level was decreased, confirming the previous results. The western blot result also showed an increased vimentin and N-cadherin level in WT-STZ groups, and both were also inhibited by GSK-J4 (Figure 2(q)).

Together, these results show that the expression of KDM6A is improved in the kidney tissues of STZ-induced DN mice and that inhibition of KDM6A mitigates kidney injury, suggesting that KDM6A can promote the progression of DN.

### 3.3. KDM6A Is an Upstream Repressor of E-cadherin Expression

**Figure 3:**
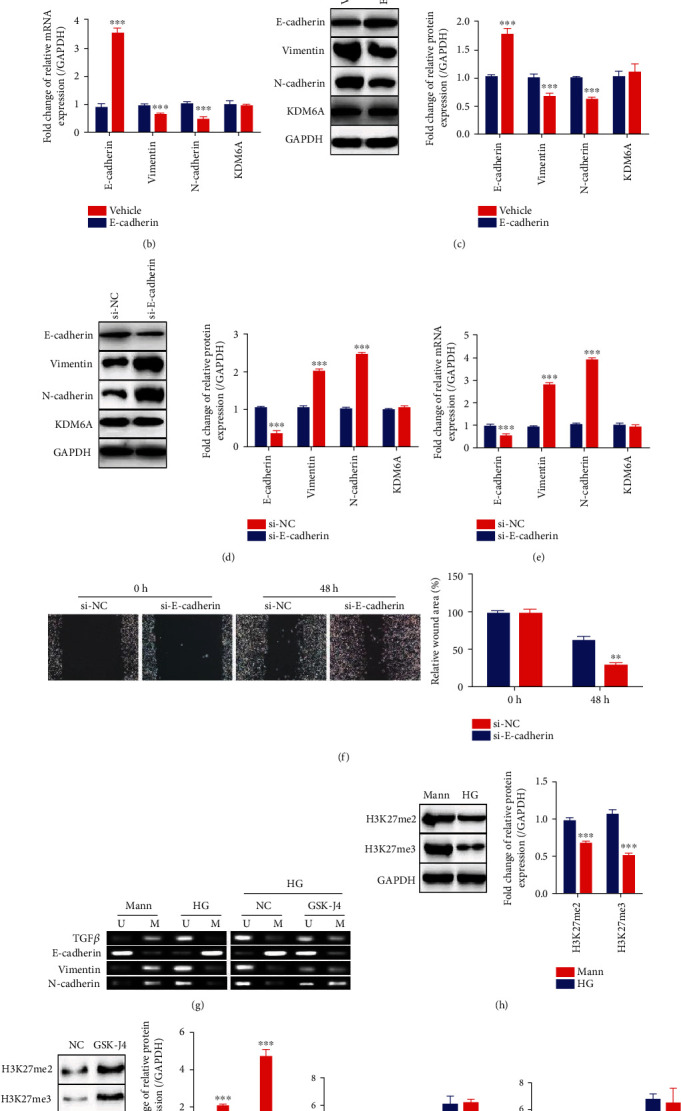
E-cadherin is a downstream target of KDM6A. (a) Representative images of migration and the graphs of wound area quantification in HK2 cells transduced with E-cadherin (HK2-E-cadherin) or empty vector (HK2-vehicle) and cultured in HG medium at 0 h and 48 h. *n* = 10. (b) The relative level of E-cadherin, vimentin, N-cadherin, and KDM6A expression by qPCR in HK2-E-cadherin and HK2-vehicle cells cultured in HG for 72 h. *n* = 3. (c) Western blot analysis of E-cadherin, vimentin, N-cadherin, and KDM6A in HK2-E-cadherin and HK2-vehicle cells cultured in HG for 72 h. *n* = 3. (d) Western blot analysis of E-cadherin, vimentin, N-cadherin, and KDM6A in HK2-E-cadherin cells with or without E-cadherin siRNA cultured in Mann for 72 h. *n* = 3. (e) The relative expression level of E-cadherin, vimentin, N-cadherin, and KDM6A by qPCR in HK2-E-cadherin cells with or without E-cadherin siRNA cultured in Mann for 72 h. *n* = 3. (f) Representative images of migration and the graphs of wound area quantification in HK2-E-cadherin cells with or without E-cadherin siRNA (si-NC and si-E-cadherin) cultured in Mann at 0 h and 48 h. *n* = 10. (g) Representative images of the methylation status analysis at TGF-*β*, E-cadherin, vimentin, and N-cadherin gene promoters in Mann- or HG-induced HK2 cell (left) and in HG-induced HK2 cells treated with or without GSK-J4 (right). *n* = 3. U: unmethylated-specific primers; M: methylated-specific primers. (h, i) Western blot analysis of H3K27me2 and H3K27me3 in Mann- or HG-induced HK2 cell (h) and in HG-induced HK2 cells treated with or without GSK-J4 (i). *n* = 3. (j, k) ChIP-qPCR for analyzing H3K27me2 and H3K27me3 binding to the E-cadherin promoter in Mann- or HG-induced HK2 cell (j) and in HG-induced HK2 cells treated with or without GSK-J4 (k). Normal IgG was used as an internal control. Mean ± standard error of the mean values is presented. ^∗∗^*P* < 0.01 and ^∗∗∗^*P* < 0.005 (Student's *t*-test).

Many reports have shown that repression of epithelial cadherin (E-cadherin) can directly trigger EMT and fibrosis [39, 40]. Based on previous works, we hypothesized that KDM6A is an upstream effector of E-cadherin and can inhibit E-cadherin expression, resulting in EMT and even fibrosis in DN. For testing our hypothesis, HK2 cells stably overexpressing E-cadherin (the HK2-E-cadherin group) were constructed and compared to vehicle-treated HK2 cells (the HK2-vehicle group). Overexpression of E-cadherin inhibited the EMT process and reduced the migration of HK2 cells compared to those in the HK2-vehicle group when cells were cultured in HG medium (Figure 3(a)). However, although E-cadherin expression was increased in the HK2-E-cadherin group, KDM6A expression did not differ between the HK2-vehicle and HK2-E-cadherin groups at either the transcriptional or translation level (Figures 3(b) and 3(c)). This finding suggests that KDM6A expression is not affected by overexpression of E-cadherin.

Furthermore, small interfering RNA targeting E-cadherin (si-E-cadherin) to knock down E-cadherin or negative control small interfering RNA (si-NC) was applied to HK2 cells cultured in the Mann medium. Notably, the expression of KDM6A remained unchanged even when EMT was aggravated by E-cadherin knockdown (Figures 3(d) and 3(e)). The migration of HK2 cells was dramatically improved with E-cadherin knockdown (Figure 3(f)).

On the other hand, we measured the methylation level of the E-cadherin promoter and other EMT-related gene promoters by MS-PCR. The methylation level in TGF-*β*, vimentin, and N-cadherin promoters was reduced, and that in the E-cadherin promoter was promoted in HG-induced HK2 cells (Figure 3(g)), which was consistent with their expression profile. However, E-cadherin promoter methylation showed a significant suppression when HG-induced HK2 cells were treated with GSK-J4, compared with the less methylation change in other gene promoters (Figure 3(g)), indicating that inhibition of KDM6A reduced E-cadherin promoter methylation, which resulted in its higher expression. The inconformity of TGF-*β*, vimentin, and N-cadherin promoter methylation and their expression for responding KDM6A inhibition also indicated that KDM6A did not regulate their expression directly.

Also, we found a decreased level of H3K27me2/3 in HG-induced HK2 cells, and both were increased by GSK-J4 (Figures 3(h) and 3(i)). According to the present cognizance, KDM6A promotes gene expression by reducing H3K27me2 or H3K27me3 level by its demethylase activity [41], which is not coincident with the profile between E-cadherin and KDM6A in our results. To further confirm, we detected the change of H3K27me2/3 binding with the E-cadherin promoter in HG-induced HK2 cells and GSK-J4-treated HK2 cells. We found that there was no significant difference between Mann-cultured and HG-cultured HK2 cells or between GKS-J4 treatment and controls in HG-induced HK2 cells (Figures 3(j) and 3(k)). It stated that the H3K27me2/3 level binding with the E-cadherin promoter displayed no obvious change during KDM6A upregulation or repression.

All these results suggest that KDM6A is an upstream regulator of E-cadherin and promotes its expression by decreasing its promoter's methylation.

### 3.4. KDM6A Is a Target of miR-199b-3p during Renal Injury

Considering the importance of miRNA in DN development, we wanted to determine whether miRNAs participate in the DN progression by targeting KDM6A mRNA. To mine potential candidate miRNAs that target KDM6A, we predicted possible miRNAs with the databases PITA, RNA22, miRmap, DIANA-microT, miRanda, PicTar, and TargetScan. Finally, we identified 8 miRNAs: hsa-miR-19a-3p, hsa-miR-19b-3p, hsa-miR-23a-3p, hsa-miR-199a-3p, hsa-miR-23b-3p, hsa-miR-199b-3p, hsa-miR-758-3p, and hsa-miR-142-3p. Each of these miRNAs was identified by at least 5 databases (Table 1). Among them, hsa-miR-142-3p, hsa-miR-19a-3p, hsa-miR-19b-3p, hsa-miR-23a-3p, and hsa-miR-23b-3p have been reported to bind to *KDM6A* in the kidney [29, 42–44]. Additionally, the fold change in hsa-miR-199a-3p, hsa-miR-199b-3p, and hsa-miR-758-3p expression between the normal kidney and DN kidney was analyzed with GEO datasets. Analysis of GSE51674, a dataset acquired from kidney fibrosis progression in human DN, showed noticeable changes in hsa-miR-199a-3p and hsa-miR-199b-3p expression but no difference in hsa-miR-758-3p expression (Figures S1A and S1B, Table S1). The change of hsa-miR-199b-3p expression was remarkable compared with that of the other 2 miRNAs.

**Figure 4:**
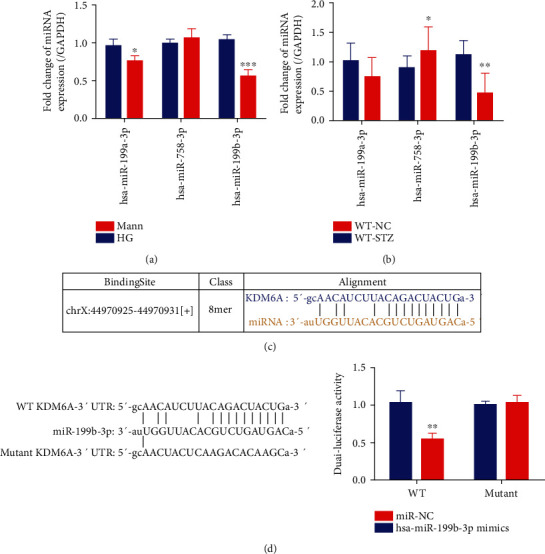
Target prediction and dual-luciferase assay between miRNAs and KDM6A. (a) The relative level of miRNA expression by qPCR in HK2 cells cultured in HG or Mann for 72 h. *n* = 3. (b) The relative expression level of miRNAs by qPCR in kidneys from C57BL/Ks mice (WT-NC) and STZ-induced mice (WT-STZ) (at 8 weeks after the onset of diabetes). *n* = 3. (c) Schematic diagram of target prediction between KDM6A and hsa-miR-199b-3p. (d) Regulation of hsa-miR-199b-3p on 3′-UTR of KDM6A in HEK293T cells by the luciferase reporter assay. *n* = 3. Mean ± standard error of the mean values is presented. ^∗^*P* < 0.05, ^∗∗^*P* < 0.01, and ^∗∗∗^*P* < 0.005 (Student's *t*-test).

For confirming the results, a qPCR assay was performed to test miRNA expression in HG-induced HK2 cells, and the results showed a trend similar to that observed in the previous work (Figure 4(a)). We also detected miRNA expression in the diabetes kidney using an STZ-induced mouse model. The results were compatible with those in HG-treated HK2 cells (Figure 4(b)).

Considering that the change in hsa-miR-199b-3p expression was the greatest, we assumed that hsa-miR-199b-3p could bind to KDM6A mRNA. Based on a predicted alignment of the binding of hsa-miR-199b-3p to KDM6A mRNA (Figure 4(c)), WT and mutant KDM6A 3′UTR luciferase plasmids, hsa-miR-199b-3p mimics, and mi-NC were designed for dual-luciferase reporter assays to confirm the prediction. Compared with cells cotransfected with WT KDM6A 3′UTR and mi-NC, cells cotransfected with WT KDM6A 3′UTR and hsa-miR-199b-3p mimics displayed a pronounced reduction in luciferase activity. However, when the WT KDM6A 3′UTR was replaced with the mutant KDM6A 3′UTR, no luciferase activity change was observed, regardless of cotransfection with hsa-miR-199b-3p mimics or mi-NC (Figure 4(d)).

These results suggest that hsa-miR-199b-3p expression is changed in the EMT of renal tubular epithelial cells and that hsa-miR-199b-3p can target *KDM6A*, demonstrating that hsa-miR-199b-3p may play a regulatory role in tubulointerstitial fibrosis in DN through downregulation of KDM6A.

### 3.5. miR-199b-3p Shows a Protective Effect against EMT of Renal Tubular Epithelial Cells through Downregulation of KDM6A

**Figure 5:**
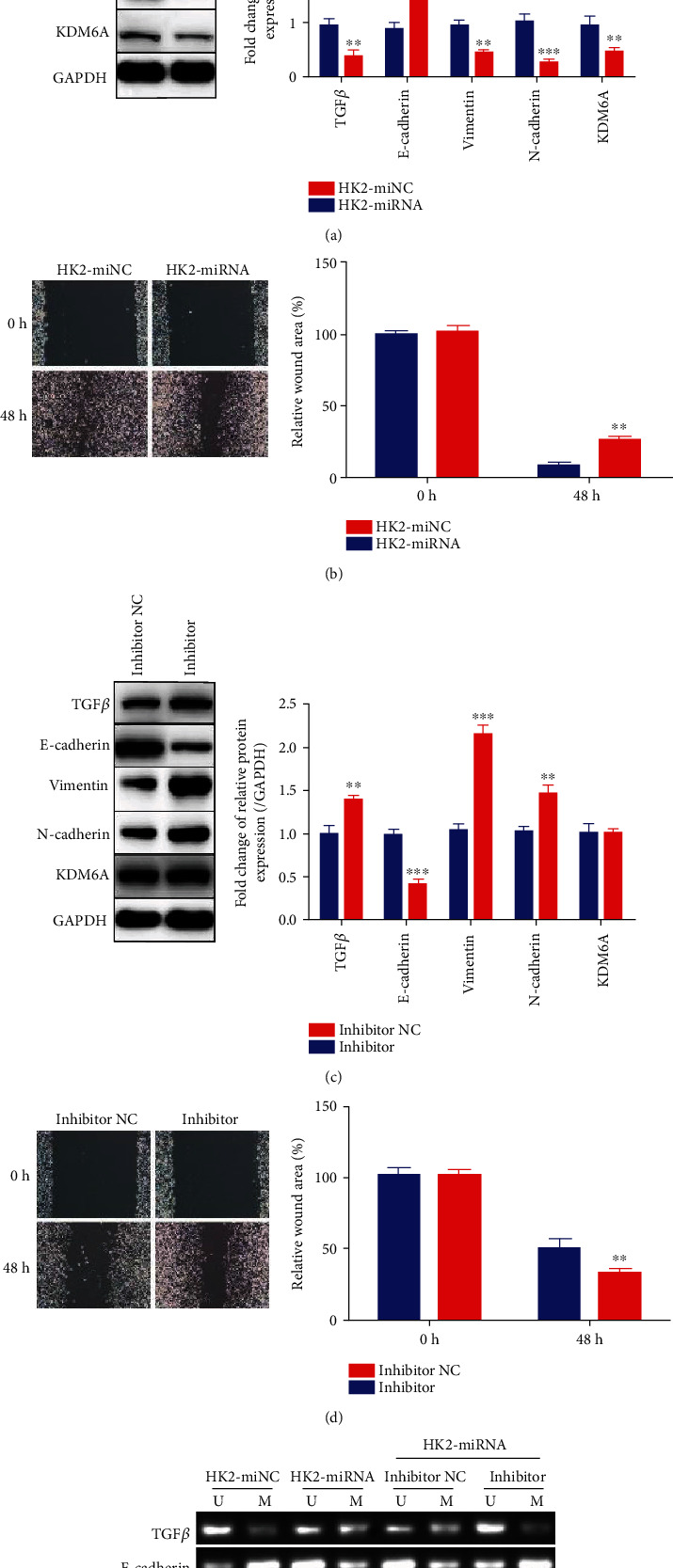
hsa-miR-199b-3p prevents the EMT of HG-induced HK2 cells through targeting KDM6A. (a) Western blot analysis of TGF*β*, E-cadherin, vimentin, N-cadherin, and KDM6A in HK2 cells transfected with mi-NC (HK2-miNC) or hsa-miR-199b-3p (HK2-miRNA) cultured in HG for 72 h. *n* = 3. (b) Representative images of migration and the graphs of wound area quantification in HK2-miNC and HK2-miRNA cells cultured in HG at 0 h and 48 h. *n* = 10. (c) Western blot analysis of TGF*β*, E-cadherin, vimentin, N-cadherin, and KDM6A in the HK2-miRNA stable cell line with negative control inhibitor (inhibitor NC) or hsa-miR-199b-3p inhibitor (inhibitor) cultured in HG for 72 h. *n* = 3. (d) Representative images of migration and the graphs of wound area quantification in inhibitor NC and inhibitor groups cultured in HG for 72 h. *n* = 10. (e) Representative images of the methylation status analysis at TGF-*β*, E-cadherin, vimentin, and N-cadherin gene promoters in HK cells transfected with mi-NC or hsa-miR-199b-3p in HG for 72 h and in the HK2-miRNA stable cell line with negative control inhibitor or hsa-miR-199b-3p inhibitor cultured in HG for 72 h. Mean ± standard error of the mean values is presented. ^∗^*P* < 0.05, ^∗∗^*P* < 0.01, and ^∗∗∗^*P* < 0.005 (Student's *t*-test).

To further explore the role of hsa-miR-199b-3p in the process of EMT in renal tubular epithelial cells, we transduced HK2 cells with lentivirus containing hsa-miR-199b-3p mimics or miR-NC and selected stable cell lines (HK2-miRNA and HK2-miNC, respectively) and cultured them in HG medium. The expression of hsa-miR-199b-3p in both cell lines was tested (Figure S2A). As the expression of hsa-miR-199b-3p in HK2-miRNA cells increased, the expression of KDM6A was inhibited in the cells, as measured by western blot analysis (Figure 5(a)), and a slightly lower transcription level of KDM6A was detected by qPCR (Figure S2A). The expression of TGF*β*, E-cadherin, vimentin, and N-cadherin was also tested. While decreased expression of TGF*β*, vimentin, and N-cadherin was observed, the E-cadherin expression was upregulated relative to that in HK2-miNC cells (Figure 5(a)). A similar tendency was also observed at the transcription level (Figure S2B). Furthermore, the HK2-miNC cells cultured in HG medium showed substantially increased EMT compared to HK2-miRNA cells cultured in HG medium with decreased migration, suggesting that hsa-miR-199b-3p could dramatically inhibit the HG-induced EMT of HK2 cells (Figure 5(b)). These results indicate that hsa-miR-199b-3p may be able to downregulate KDM6A and further protects HK2 cells from HG-induced injury.

To confirm the influence of hsa-miR-199b-3p on HK2 cells through its downregulation of KDM6A, we transfected HK2-miRNA cells with an hsa-miR-199b-3p inhibitor or inhibitor NC to determine whether inhibition of hsa-miR-199b-3p could exacerbate HG-induced EMT. The qPCR results showed the successful inhibition of hsa-miR-199b-3p due to the inhibitor, accompanied by the restored expression of KDM6A (Figure S2C). Consistently, expression of EMT markers, at both the mRNA and protein levels, showed a tendency of E-cadherin decreasing and others increasing (Figure 5(c), Figure S2D). As expected, the expression of KDM6A at the translation level was indeed restored due to the inhibitor presence (Figure 5(c)). In the presence of the inhibitor, the migration of HK2-miRNA cells was increased, which indicated that HK2-miRNA cells exhibited a more mesenchymal-like phenotype than cells in the inhibitor NC group did (Figure 5(d)). These results suggest that the restoration of KDM6A expression due to the repression of hsa-miR-199b-3p can aggravate the EMT process.

We also measured the methylation in TGF*β*, E-cadherin, vimentin, and N-cadherin promoters by MS-PCR. It was easily observed that the methylation in the E-cadherin promoter was inhibited in the presence of hsa-miR-199b-3p while promoted by the inhibitor (Figure 5(e)).

Together, these results show that hsa-miR-199b-3p can reduce EMT in renal tubular epithelial cells via suppressing KDM6A expression.

### 3.6. miR-199b-3p Could Inhibit Kidney Injury Caused by DN via Regulating KDM6A Expression

**Figure 6:**
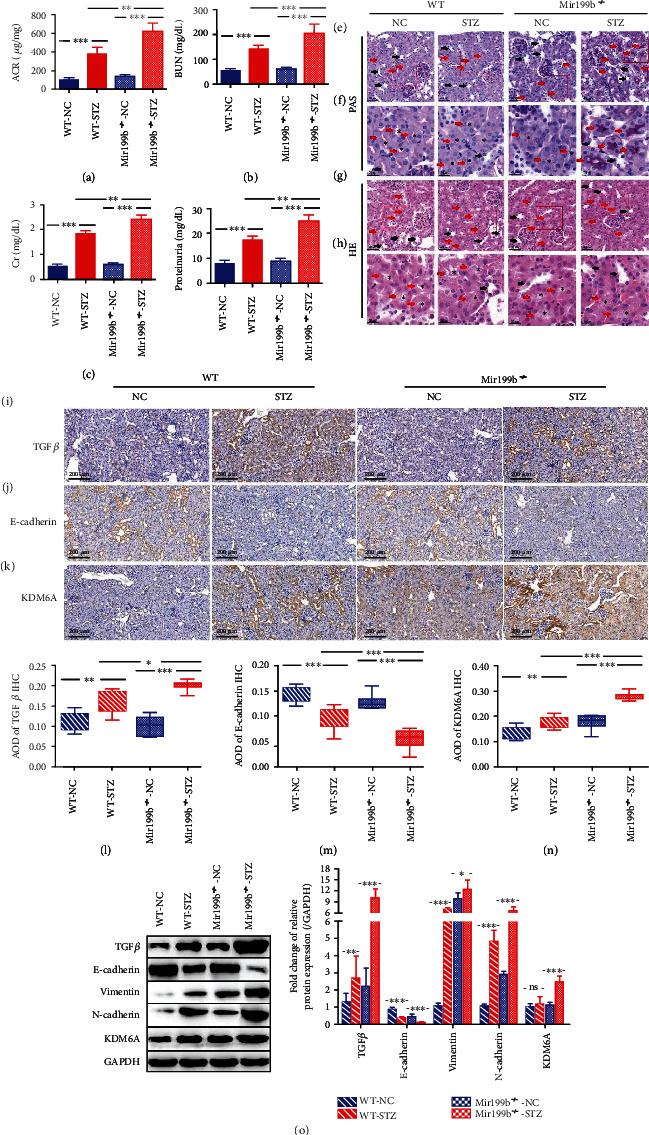
Knockout of miR-199b-3p aggravated renal dysfunction in mice. (a–d) The graphs showing the results of ACR, BUN, Cr, and proteinuria in different treated mice at 8 weeks after the onset of diabetes. C57BL/6 mice without any treatment (WT-NC), C57BL/6 mice treated with STZ (WT-STZ), Mir199b-knockout C57BL/6 mice without any treatment (Mir199b^−/−^-NC), and Mir199b-knockout C57BL/6 mice treated with STZ (Mir199b^−/−^-STZ). *n* = 6. (e, g) Representative images of PAS staining and HE staining in kidneys from different mice with different treatments. Scale bar, 40 *μ*m. Proximal tubule marked with red arrows and distal tubule marked with black arrows. (f, h) Representative images of area amplification marked with red boxes in (e) and (g), respectively. Scale bar, 20 *μ*m. Proximal tubule marked with red arrows and distal tubule marked with black arrows. Lumen of proximal tubules marked with black asterisk. (i–k) Representative images of TGF*β*, E-cadherin, and KDM6A immunohistochemical staining in kidneys from mice with different treatments. Scale bar, 200 *μ*m. (l–n) The graphs showing quantification (AOD = IOD/area) of TGF*β*, E-cadherin, and KDM6A immunohistochemical staining results in (i)–(k), respectively. *n* = 10. (o) Western blot analysis of TGF*β*, E-cadherin, vimentin, N-cadherin, and KDM6A in the kidney from mice. *n* = 6. Mean ± standard error of the mean values is presented. ^∗^*P* < 0.05, ^∗∗^*P* < 0.01, and ^∗∗∗^*P* < 0.005 (one-way ANOVA).

To further confirm the function of miR-199b-3p *in vivo*, we induced DN in Mir199b-deficient mice (Mir199b^−/−^, Figure S3A) using STZ and used another group of mice as a negative control group (the Mir199b^−/−^-STZ and Mir199b^−/−^-NC groups, respectively). Several typical pathological characteristics of DN were measured in the different groups, and all of these markers showed a similar tendency. The ACR, BUN, Cr, and proteinuria levels in the Mir199b^−/−^-STZ group were significantly greater than those in the WT-STZ group, indicating the elimination of the protective function of mmu-miR-199b-3p due to Mir199b deficiency (Figures 6(a)–6(d)). Furthermore, the histological pathological features, such as reduced proximal tubule lumen, progressive renal proximal tubular dilatation, and thickened and irregular tubular basement membrane, of the kidneys from WT and Mir199b^−/−^ STZ-induced diabetic mice were observed through HE and PAS staining. With STZ-mediated induction of diabetes, significantly increased renal proximal tubular injury emerged in Mir199b^−/−^ mice compared to WT mice with no significant difference in the distal tubule and glomerulus (Figures 6(e)–6(h)). Compared with the WT-STZ group, the Mir199b^−/−^-STZ group showed increased expression of TGF*β*, vimentin, and N-cadherin and decreased expression of E-cadherin by IHC, western blot, and qPCR, which was consistent with the pathological results (Figures 6(i), 6(j), 6(l), 6(m), and 6(o), Figure S3B). However, more KDM6A was expressed in diabetic kidney tissues in the absence of mmu-miR-199b-3p at both the transcriptional and translational levels (Figures 6(k), 6(n), and 6(o), Figure S3B).

In summary, based on these *in vivo* results, we find that the absence of mmu-miR-199b-3p is closely associated with the exacerbation of kidney injury in STZ-induced diabetic mice by removing the repression of KDM6A, indicating that mmu-miR-199b-3p plays a protective role against kidney injury in DN through downregulating KDM6A.

## 4. Discussion

Despite the numerous studies on DN, therapeutic interventions to prevent DN remain insufficient, indicating that potential pathways that participate in the progression of DN have yet to be explored. Here, our study demonstrates that upregulation of KDM6A in the diabetic kidney can inhibit E-cadherin expression and trigger EMT and dysfunction in the renal tubule in the diabetic kidney, eventually leading to DN. However, we identified a new role for miR-199b-3p in DN, which protects the kidney from diabetic-induced injury by repressing KMD6A expression.

Many studies have suggested that EMT plays an important role in renal fibrosis. It is associated with tubule dysfunctions and tubular injury and is a pathological feature of kidney diseases, including DN [9–12]. In the present work, we found that KDM6A promotes the EMT progression in HK2 cells induced by HG. Similarly, the positive role of KMD6A in regulating the migration and invasion of hematopoietic stem cells and breast cancer cells, usually caused by EMT, was found, indicating that KMD6A might positively regulate EMT in those cell types [45, 46]. The results of our *in vivo* experiments revealed that inhibition of KDM6A decreases the degree of EMT and tubular injury in the diabetic kidney and eases the symptoms of kidney injury. Coincidentally, Lin et al. also found that suppression of KDM6A in STZ-induced mice had a protective effect on kidney injury. They further demonstrated that KDM6A aggravated DN by disturbing podocyte function by increasing KLF10, which could inhibit nephrin expression [21]. Chen et al. showed that overexpression of KDM6A accelerated the progression of diabetic kidney diseases [20]. On the other hand, the role of KDM6A in EMT progression remains controversial. Van den Beucken et al. showed that inhibition of KDM6A promoted EMT and cancer stemness [47], and Zhou et al. showed that downregulation of KDM6A repressed E-cadherin expression [48], which could be attributed to increased histone H3K27 methylation. Considering the multiple functions of KDM6A during EMT in different diseases and cell types and the methylation change of the E-cadherin promoter in our results, KDM6A may regulate the EMT process via many different pathways. Interestingly, we found that the E-cadherin's expression, a critical regulatory gene of EMT, was decreased, and its promoter methylation was upregulated with increasing KDM6A expression. In contrast, the level of H3K27me2/3 binding with the E-cadherin promoter displayed no significant variation. Therefore, we assume a transcription factor “X” between KDM6A and E-cadherin responsible for suppressing E-cadherin. KDM6A upregulates the expression of “X” by reducing H3K27me3, resulting in E-cadherin inhibition. Like Lin et al.'s work, KDM6A repressed nephrin expression by increasing KLF10 and aggravated diabetic podocyte dysfunction [21].

Many studies have shown that hsa-miR-199b-3p is expressed in many tissues and plays a functional role in different cancers. Graham et al. showed that hsa-miR-199b-3p was dramatically downregulated in the sera of patients with papillary thyroid cancer [49]. Wang et al. found that hsa-miR-199b-3p is a key miRNA related to hepatocellular carcinoma [50]. Koshizuka et al. showed that hsa-miR-199b-3p inhibits cancer cell migration and invasion in head and neck cancer by regulating ITGA3 [26]. Similarly, Sakaguchi et al. also found that hsa-miR-199b-3p functions as a tumor suppressor in bladder cancer by targeting ITGA3 [51]. In addition to cancer, hsa-miR-199b-3p plays an important role in other diseases. The apoptosis of cerebral microvascular endothelial cells was repressed via upregulation of hsa-miR-199b-3p in ischemic stroke [28]. Wu et al. showed that hsa-miR-199b-3p was substantially increased in extracellular vesicles from the nasal mucus of patients with allergic rhinitis [52]. Dolz et al. also found that hsa-miR-199b-3p exhibited remarkably higher expression in patients with asymptomatic carotid artery stenosis progression [53]. However, the role of hsa-miR-199b-3p in DN is unclear. As described in this study, KDM6A is regulated by several miRNAs in the kidney tissue. For the first time, we have defined KDM6A as a target of hsa-miR-199b-3p in the diabetic kidney, which may provide new insights into the mechanisms by which DN progresses.

Although hsa-miR-199b-3p was proven to relieve diabetes-induced kidney injury by inhibiting KDM6A expression, its upregulation was observed in HG-induced HK2 cells and the kidney tissues of STZ-induced mice. We think that it may be feedback or compensation for KDM6A increase. Similar physiological characteristics could be found elsewhere. Another might be that there are several signaling pathways for inducing a higher level of KDM6A in renal epithelial cells even with the upregulation of miR-199b-3p, which might be consistent with the assumption that upregulation of miR-199b-3p is a compensation.

## 5. Conclusion

Here, we provide evidence that KDM6A promotes the EMT, injury, and dysfunction of the renal tubule in DN by downregulating E-cadherin expression, eventually resulting in severe kidney injury, while hsa-miR-199b-3p rescues this renal damage by repressing KDM6A expression. In the future, hsa-miR-199b-3p may be used as a biomarker or target for the diagnosis or treatment of DN.

## Figures and Tables

**Table 1:** The predictive results of miRNA targeting KDM6A.

miRNA ID	miRNA name	Databases							Numbers of databases
		PITA	RNA22	miRmap	microT	miRanda	PicTar	TargetScan	
MIMAT0000073	hsa-miR-19a-3p	√	×	×	√	√	√	√	5
MIMAT0000074	hsa-miR-19b-3p	√	×	×	√	√	√	√	5
MIMAT0000078	hsa-miR-23a-3p	√	×	×	√	√	√	√	5
MIMAT0000232	hsa-miR-199a-3p	√	×	√	√	×	√	√	5
MIMAT0000418	hsa-miR-23b-3p	√	×	×	√	√	√	√	5
MIMAT0000434	hsa-miR-142-3p	√	×	√	√	√	√	√	6
MIMAT0003879	hsa-miR-758-3p	√	×	√	√	√	√	√	6
MIMAT0004563	hsa-miR-199b-3p	√	×	√	√	×	√	√	5

## Data Availability

The datasets supporting the conclusions of this article are included within the article and its additional files.

## Supplementary Materials

Supplementary Materials Supplementary Figure 1: the analyzed results from GEO datasets. (A) The expression profile of has-miR-199a-3p/has-miR-199b-3p in healthy persons and patients with DN from dataset GSE51674 in GEO datasets. (B) The logFC of miRNA expression analyzed in patients with DN from dataset GSE51674 compared with healthy persons. Supplementary Figure 2: the mRNA expression profile of KDM6A, TGF*β*, and E-cadherin with the change in miR-199b-3p expression. (A) The relative expression level of has-miR-199b-3p and KDM6A in HK2 cells with mi-NC (HK2-miNC) or has-miR-199b-3p (HK2-miRNA) overexpression cultured in HG for 72 h. *n* = 3. (B) The relative expression level of TGF*β*, E-cadherin, vimentin, and N-cadherin in HK2-miNC and HK2-miRNA cells cultured in HG for 72 h. *n* = 3. (C) The relative level of has-miR-199b-3p and KDM6A expression in HK2-miRNA cells with negative control siRNA (inhibitor NC) or miR-199b-3p inhibitor (inhibitor) cultured in HG for 72 h. *n* = 3. (D) The relative expression level of TGF*β*, E-cadherin, vimentin, and N-cadherin in HK2-miRNA cells with inhibitor NC or inhibitor cultured in HG for 72 h. *n* = 3. Mean ± standard error of the mean values is presented. ^∗^*P* < 0.05, ^∗∗^*P* < 0.01, and ^∗∗∗^*P* < 0.005 (Student's *t*-test). Supplementary Figure 3: the mRNA expression of KDM6A, TGF*β*, and E-cadherin in Mir199b-knockout mice. (A) The relative level of mmu-miR-199b-3p expression in kidney tissue from different mice. C57BL/Ks mice (WT) and Mir199b-knockout C57BL/Ks mice (Mir199b^−/−^). *n* = 3. (B) The relative expression level of TGF*β*, E-cadherin, vimentin, N-cadherin, and KDM6A in kidney tissue from mice with different treatments at 8 weeks after the onset of diabetes. C57BL/Ks mice without any treatment (WT-NC), C57BL/Ks mice treated with STZ (WT-STZ), Mir199b-knockout C57BL/Ks mice without any treatment (Mir199b^−/−^-NC), and Mir199b-knockout C57BL/Ks mice treated with STZ (Mir199b^−/−^-STZ). *n* = 3. Mean ± standard error of the mean values is presented. ^∗∗^*P* < 0.01 and ^∗∗∗^*P* < 0.005 (Student's *t*-test in A; one-way ANOVA in B). Table S1: the analysis of the miRNA expression profile from the GSE51674 dataset. Table S2: primers, siRNA, and miRNA used in this study.
