# Supplementary figures and images for: hsa-miR-199b-3p Prevents the Epithelial-Mesenchymal Transition and Dysfunction of the Renal Tubule by Regulating E-cadherin through Targeting KDM6A in Diabetic Nephropathy
Source: Oxid Med Cell Longev. 2021 Jun 27;2021:8814163. doi: 10.1155/2021/8814163 (PMC8257373; doi:10.1155/2021/8814163)

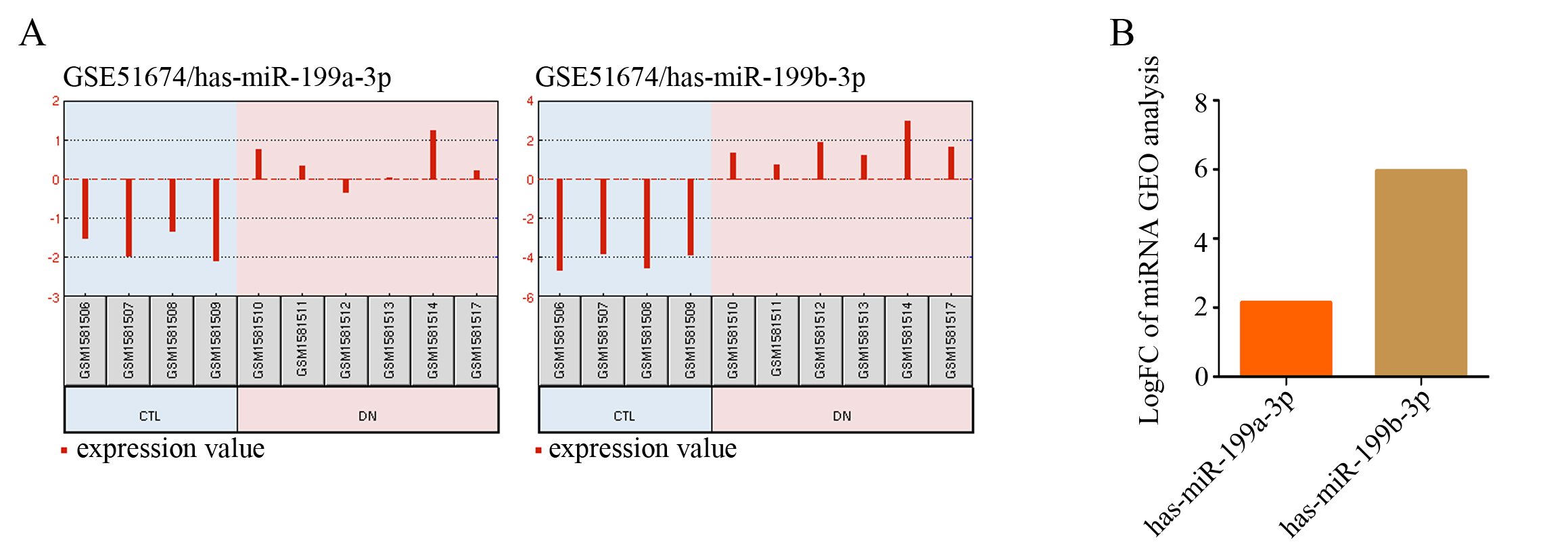

Supplement: Supplementary Materials — Supplementary Figure 1: the analyzed results from GEO datasets. (A) The expression profile of has-miR-199a-3p/has-miR-199b-3p in healthy persons and patients with DN from dataset GSE51674 in GEO datasets. (B) The logFC of miRNA expression analyzed in patients with DN from dataset GSE51674 compared with healthy persons. Supplementary Figure 2: the mRNA expression profile of KDM6A, TGFβ, and E-cadherin with the change in miR-199b-3p expression. (A) The relative expression level of has-miR-199b-3p and KDM6A in HK2 cells with mi-NC (HK2-miNC) or has-miR-199b-3p (HK2-miRNA) overexpression cultured in HG for 72 h. n = 3. (B) The relative expression level of TGFβ, E-cadherin, vimentin, and N-cadherin in HK2-miNC and HK2-miRNA cells cultured in HG for 72 h. n = 3. (C) The relative level of has-miR-199b-3p and KDM6A expression in HK2-miRNA cells with negative control siRNA (inhibitor NC) or miR-199b-3p inhibitor (inhibitor) cultured in HG for 72 h. n = 3. (D) The relative expression level of TGFβ, E-cadherin, vimentin, and N-cadherin in HK2-miRNA cells with inhibitor NC or inhibitor cultured in HG for 72 h. n = 3. Mean ± standard error of the mean values is presented. ∗P < 0.05, ∗∗P < 0.01, and ∗∗∗P < 0.005 (Student's t-test). Supplementary Figure 3: the mRNA expression of KDM6A, TGFβ, and E-cadherin in Mir199b-knockout mice. (A) The relative level of mmu-miR-199b-3p expression in kidney tissue from different mice. C57BL/Ks mice (WT) and Mir199b-knockout C57BL/Ks mice (Mir199b−/−). n = 3. (B) The relative expression level of TGFβ, E-cadherin, vimentin, N-cadherin, and KDM6A in kidney tissue from mice with different treatments at 8 weeks after the onset of diabetes. C57BL/Ks mice without any treatment (WT-NC), C57BL/Ks mice treated with STZ (WT-STZ), Mir199b-knockout C57BL/Ks mice without any treatment (Mir199b−/−-NC), and Mir199b-knockout C57BL/Ks mice treated with STZ (Mir199b−/−-STZ). n = 3. Mean ± standard error of the mean values is presented. ∗∗P < 0.01 and ∗∗∗P < 0.005 [file 8814163.f1.zip › 8814163.f1/Figure S1 (1).jpg]

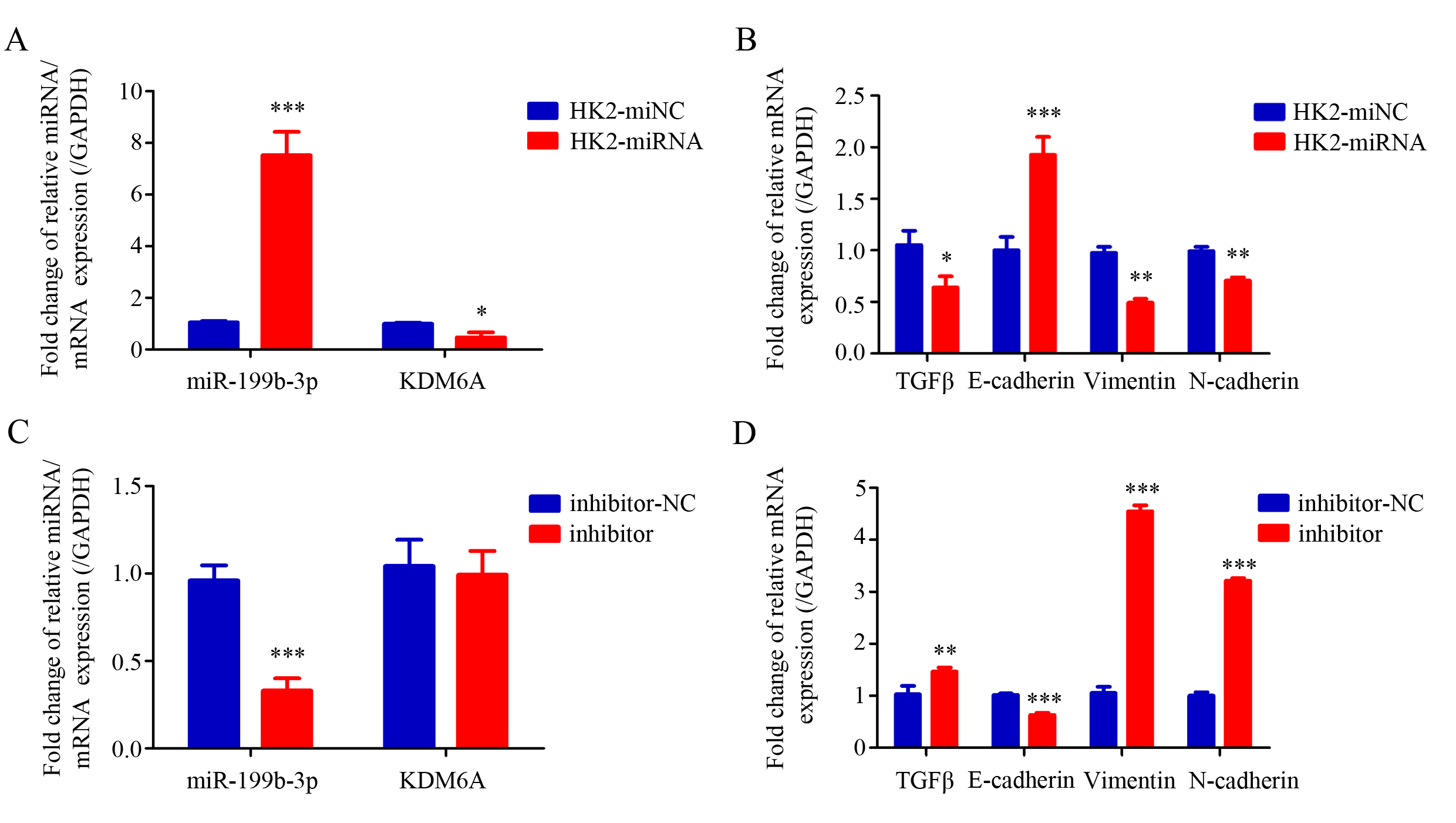

Supplement: Supplementary Materials — Supplementary Figure 1: the analyzed results from GEO datasets. (A) The expression profile of has-miR-199a-3p/has-miR-199b-3p in healthy persons and patients with DN from dataset GSE51674 in GEO datasets. (B) The logFC of miRNA expression analyzed in patients with DN from dataset GSE51674 compared with healthy persons. Supplementary Figure 2: the mRNA expression profile of KDM6A, TGFβ, and E-cadherin with the change in miR-199b-3p expression. (A) The relative expression level of has-miR-199b-3p and KDM6A in HK2 cells with mi-NC (HK2-miNC) or has-miR-199b-3p (HK2-miRNA) overexpression cultured in HG for 72 h. n = 3. (B) The relative expression level of TGFβ, E-cadherin, vimentin, and N-cadherin in HK2-miNC and HK2-miRNA cells cultured in HG for 72 h. n = 3. (C) The relative level of has-miR-199b-3p and KDM6A expression in HK2-miRNA cells with negative control siRNA (inhibitor NC) or miR-199b-3p inhibitor (inhibitor) cultured in HG for 72 h. n = 3. (D) The relative expression level of TGFβ, E-cadherin, vimentin, and N-cadherin in HK2-miRNA cells with inhibitor NC or inhibitor cultured in HG for 72 h. n = 3. Mean ± standard error of the mean values is presented. ∗P < 0.05, ∗∗P < 0.01, and ∗∗∗P < 0.005 (Student's t-test). Supplementary Figure 3: the mRNA expression of KDM6A, TGFβ, and E-cadherin in Mir199b-knockout mice. (A) The relative level of mmu-miR-199b-3p expression in kidney tissue from different mice. C57BL/Ks mice (WT) and Mir199b-knockout C57BL/Ks mice (Mir199b−/−). n = 3. (B) The relative expression level of TGFβ, E-cadherin, vimentin, N-cadherin, and KDM6A in kidney tissue from mice with different treatments at 8 weeks after the onset of diabetes. C57BL/Ks mice without any treatment (WT-NC), C57BL/Ks mice treated with STZ (WT-STZ), Mir199b-knockout C57BL/Ks mice without any treatment (Mir199b−/−-NC), and Mir199b-knockout C57BL/Ks mice treated with STZ (Mir199b−/−-STZ). n = 3. Mean ± standard error of the mean values is presented. ∗∗P < 0.01 and ∗∗∗P < 0.005 [file 8814163.f1.zip › 8814163.f1/Figure S2 (1).jpg]

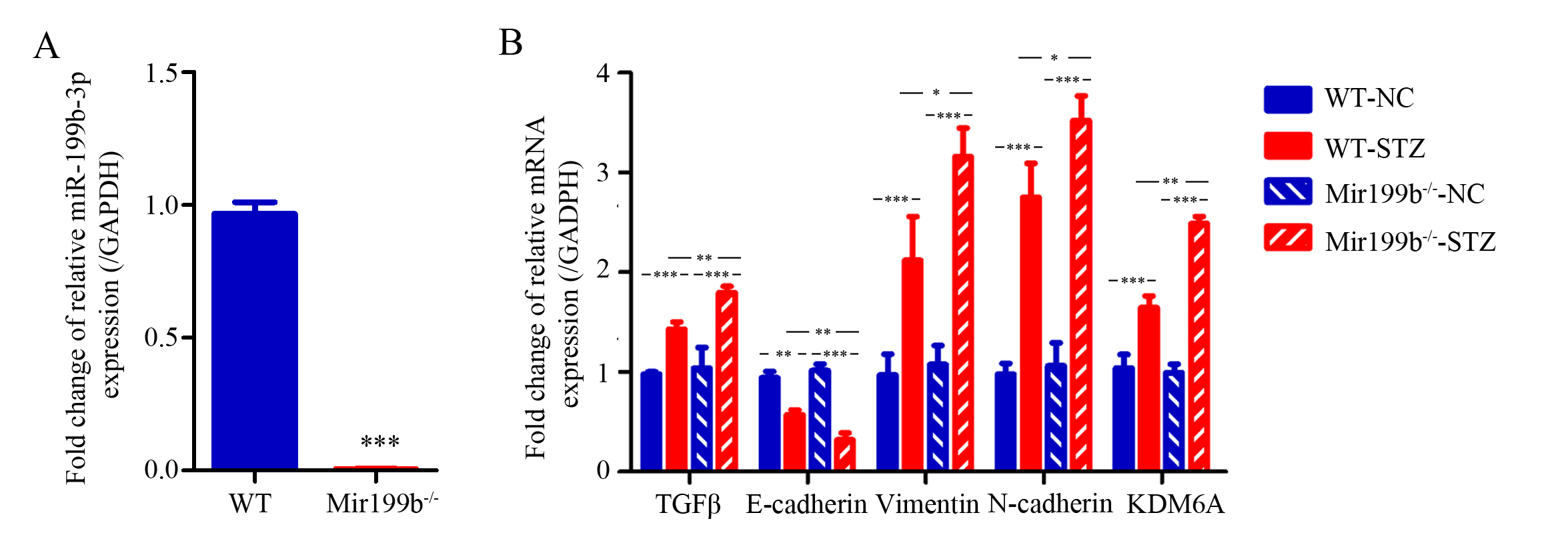

Supplement: Supplementary Materials — Supplementary Figure 1: the analyzed results from GEO datasets. (A) The expression profile of has-miR-199a-3p/has-miR-199b-3p in healthy persons and patients with DN from dataset GSE51674 in GEO datasets. (B) The logFC of miRNA expression analyzed in patients with DN from dataset GSE51674 compared with healthy persons. Supplementary Figure 2: the mRNA expression profile of KDM6A, TGFβ, and E-cadherin with the change in miR-199b-3p expression. (A) The relative expression level of has-miR-199b-3p and KDM6A in HK2 cells with mi-NC (HK2-miNC) or has-miR-199b-3p (HK2-miRNA) overexpression cultured in HG for 72 h. n = 3. (B) The relative expression level of TGFβ, E-cadherin, vimentin, and N-cadherin in HK2-miNC and HK2-miRNA cells cultured in HG for 72 h. n = 3. (C) The relative level of has-miR-199b-3p and KDM6A expression in HK2-miRNA cells with negative control siRNA (inhibitor NC) or miR-199b-3p inhibitor (inhibitor) cultured in HG for 72 h. n = 3. (D) The relative expression level of TGFβ, E-cadherin, vimentin, and N-cadherin in HK2-miRNA cells with inhibitor NC or inhibitor cultured in HG for 72 h. n = 3. Mean ± standard error of the mean values is presented. ∗P < 0.05, ∗∗P < 0.01, and ∗∗∗P < 0.005 (Student's t-test). Supplementary Figure 3: the mRNA expression of KDM6A, TGFβ, and E-cadherin in Mir199b-knockout mice. (A) The relative level of mmu-miR-199b-3p expression in kidney tissue from different mice. C57BL/Ks mice (WT) and Mir199b-knockout C57BL/Ks mice (Mir199b−/−). n = 3. (B) The relative expression level of TGFβ, E-cadherin, vimentin, N-cadherin, and KDM6A in kidney tissue from mice with different treatments at 8 weeks after the onset of diabetes. C57BL/Ks mice without any treatment (WT-NC), C57BL/Ks mice treated with STZ (WT-STZ), Mir199b-knockout C57BL/Ks mice without any treatment (Mir199b−/−-NC), and Mir199b-knockout C57BL/Ks mice treated with STZ (Mir199b−/−-STZ). n = 3. Mean ± standard error of the mean values is presented. ∗∗P < 0.01 and ∗∗∗P < 0.005 [file 8814163.f1.zip › 8814163.f1/Figure S3 (1).jpg]
